# Supplementary material for: Chemical Composition of Green Pea (Pisum sativum L.) Pods Extracts and Their Potential Exploitation as Ingredients in Nutraceutical Formulations
Source: Antioxidants (Basel). 2021 Dec 31;11(1):105. doi: 10.3390/antiox11010105 (PMC8772770; doi:10.3390/antiox11010105)
Supplement: Supplementary file 1 [file antioxidants-11-00105-s001.zip › antioxidants-1513185-supplementary.pdf]

Supplementary Materials:

Table S1. Spectrometric parameters of the studied analytes.

Compound	RT (min)	Adduct Ion	Chemical Formula	Theoretical Mass (m/z)	Measured Mass (m/z)	Accuracy (Δ mg/kg)	LOD (mg/kg)	LOQ (mg/kg)
Quinic acid	0.31	[M-H]-	$C_7H_{12}O_6$	191.05531	191.05611	4.18727	0.019	0.057
Gallic acid	0.82	[M-H]-	$C_7H_6O_5$	169.01425	169.01490	3.84583	0.039	0.117
Protocatechuic acid	1.57	[M-H]-	$C_7H_6O_4$	153.01930	153.01857	-4.77064	0.019	0.057
Epicatechin	3.09	[M-H]-	$C_{15}H_{14}O_7$	289.07176	289.07202	0.89943	0.019	0.057
5-caffeoylquinic acid	3.18	[M-H]-	$C_{16}H_{18}O_9$	353.08780	353.08798	0.50979	0.019	0.057
Catechin	3.27	[M-H]-	$C_{15}H_{14}O_6$	289.07175	289.07205	1.03780	0.039	0.117
p-Coumaric acid	3.37	[M-H]-	$C_9H_8O_3$	163.04001	163.03937	-3.92542	0.019	0.057
Ferulic acid	3.46	[M-H]-	$C_{10}H_{10}O_4$	193.05063	193.05016	-2.43459	0.039	0.117
Genistein	3.49	[M-H]-	$C_{15}H_{10}O_5$	269.04554	269.04562	0.29735	0.019	0.057
Naringin	3.56	[M-H]-	$C_{27}H_{32}O_{14}$	579.17193	579.17212	0.32805	0.019	0.057
Rutin	3.60	[M-H]-	$C_{27}H_{30}O_{16}$	609.14611	609.14673	1.01782	0.019	0.057
Quercetin 3 galattoside	3.60	[M-H]-	$C_{21}H_{20}O_{12}$	463.08820	463.08817	-0.06478	0.039	0.117
Rosamarinic acid	3.62	[M-H]-	$C_{18}H_{16}O_8$	359.07724	359.07697	-0.75193	0.019	0.057
Kaempferol 3 glucoside	3.63	[M-H]-	$C_{21}H_{20}O_{11}$	447.09195	447.09329	2.99715	0.019	0.057
Apigenin	3.67	[M-H]-	$C_{15}H_{10}O_5$	269.04555	269.04556	0.03717	0.039	0.117
Apigenin-7-O-glucoside	3.68	[M-H]-	$C_{21}H_{20}O_{10}$	431.09837	431.09875	-1.07788	0.019	0.057
Diosmin	3.69	[M-H]-	$C_{28}H_{31}O_{15}$	607.16684	607.16534	-2.47049	0.019	0.057
Isorhamnetin 3 rutinoside	3.71	[M-H]-	$C_{28}H_{32}O_{16}$	623.16176	623.16174	-0.03209	0.019	0.057
Myricetin	3.73	[M-H]-	$C_{14}H_{10}O8$	317.03029	317.02924	-3.31199	0.019	0.057
Daidzein	3.76	[M-H]-	$C_{15}10_0O_4$	253.05063	253.05035	-1.10650	0.019	0.057
Hesperidin	3.79	[M-H]-	$C_{27}H_{30}O_{16}$	609.14611	609.14612	0.01642	0.019	0.057
Quercetin	3.87	[M-H]-	$C_{15}H_{10}O_7$	301.03538	301.03508	-0.99656	0.019	0.057
Naringenin	3.91	[M-H]-	$C_{15}H_{12}O_5$	271.06120	271.06110	-0.36892	0.019	0.057
Luteolin	3.95	[M-H]-	$C_{15}H_{10}O_6$	285.04046	285.04086	1.40331	0.039	0.117

Abbreviations: LOD: limit of detection; LOQ: limit of quantification.

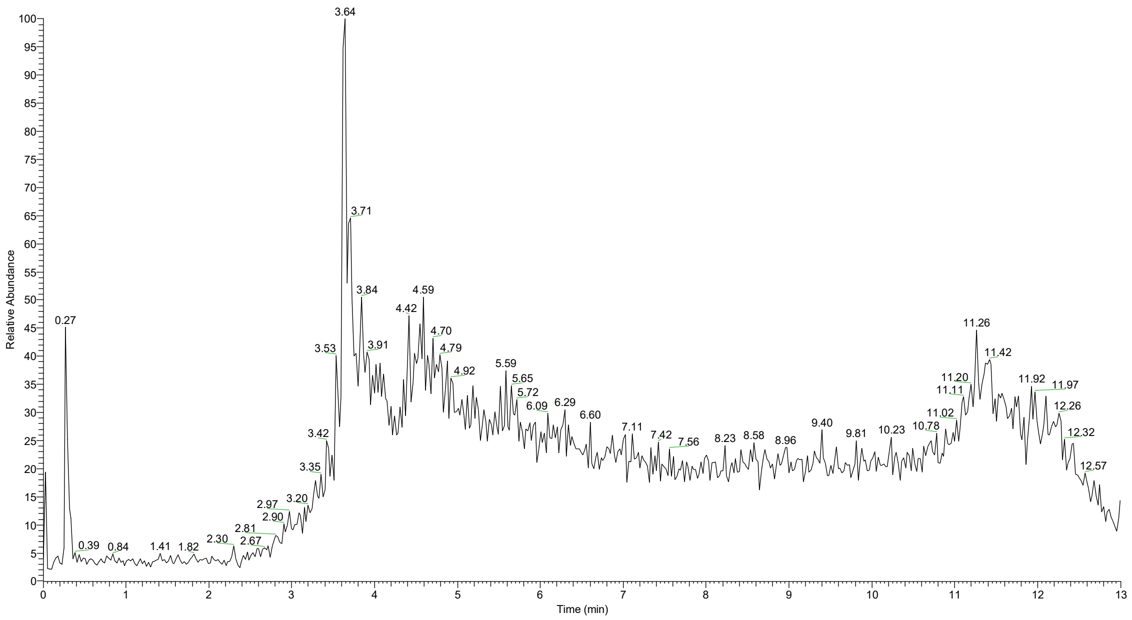

Figure S1. Total Ion Chromatogram (TIC) obtained through UHPLC-Q-Orbitrap HRMS analysis

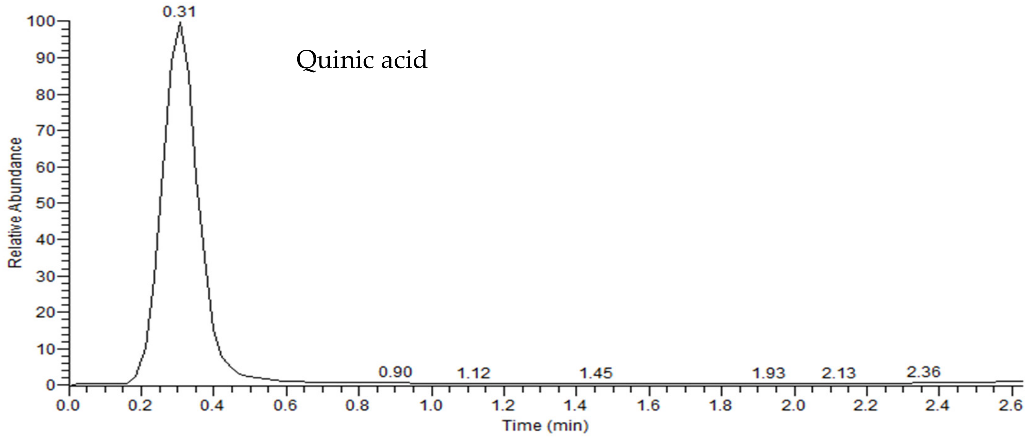

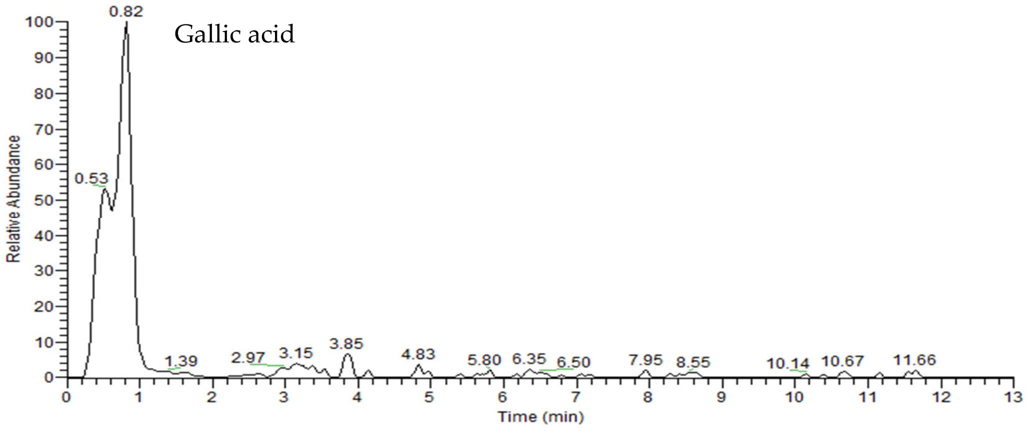

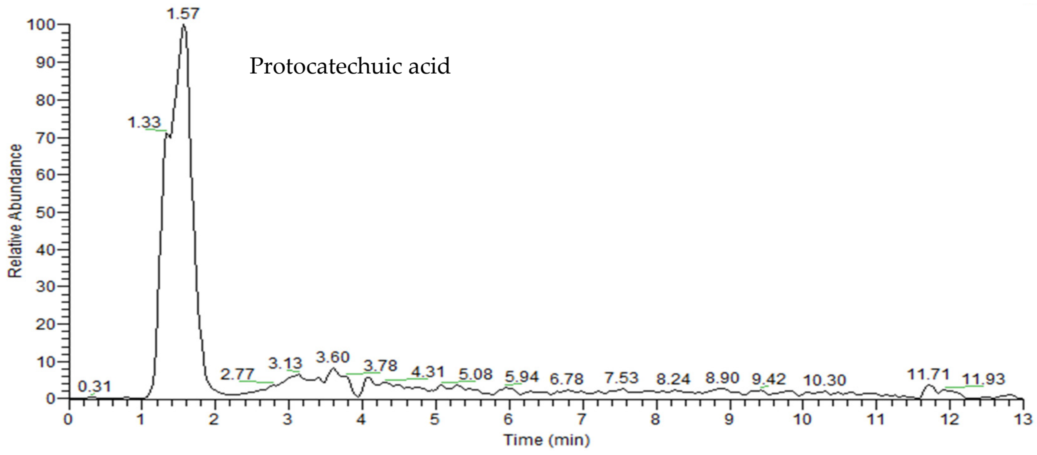
Protocatechuic acid
Relative Abundance
Time (min)

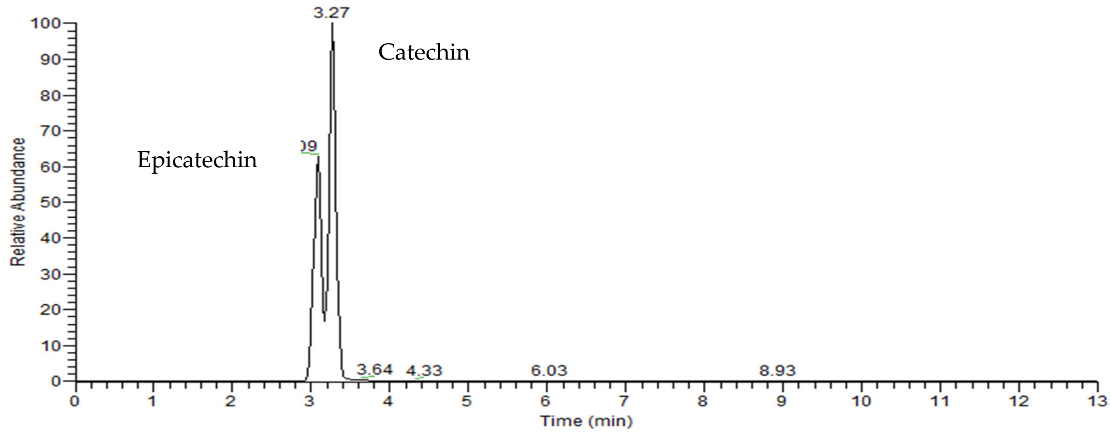
Catechin
Epicatechin
Relative Abundance
Time (min)

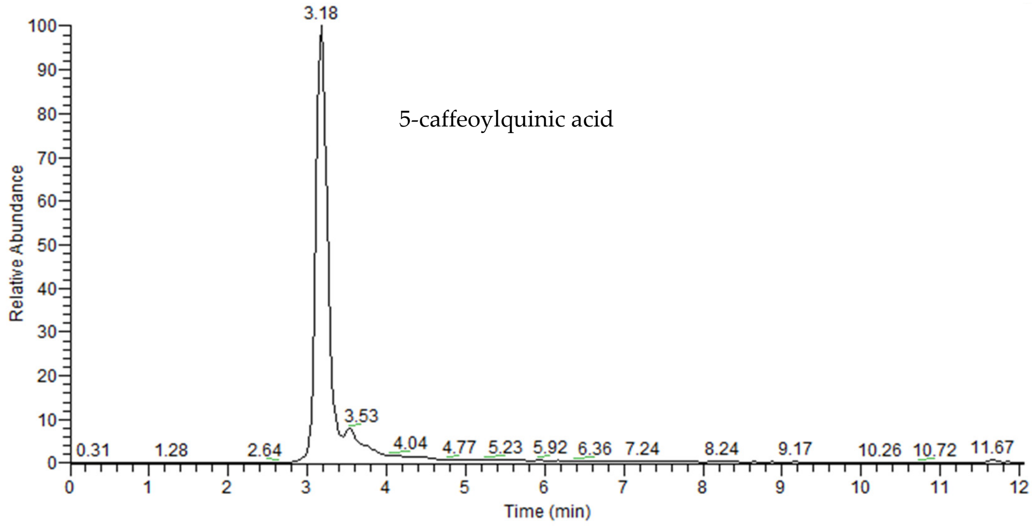
5-caffeoylquinic acid
Relative Abundance
Time (min)

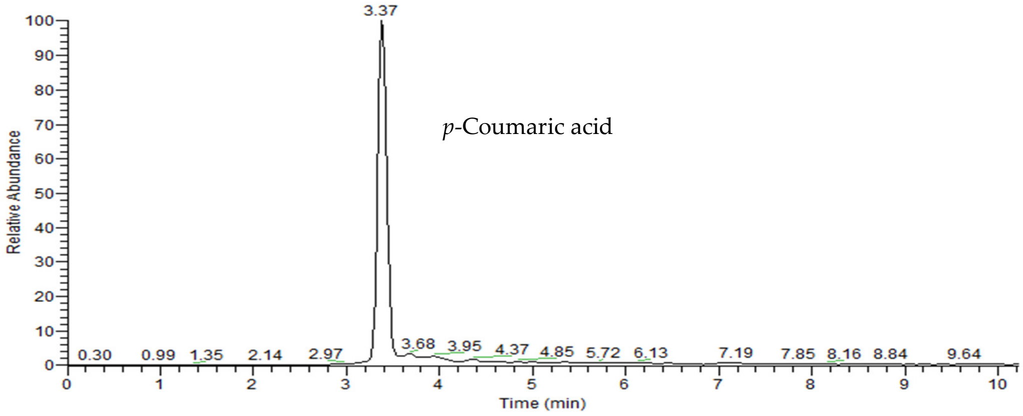
3.37
p-Coumaric acid
Relative Abundance
Time (min)
0.30 0.99 1.35 2.14 2.97 3.68 3.95 4.37 4.85 5.72 6.13 7.19 7.85 8.16 8.84 9.64

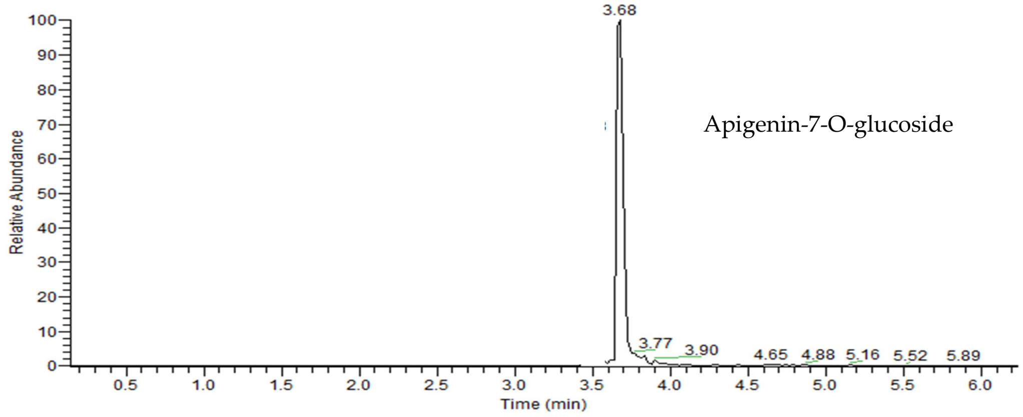
3.68
Apigenin-7-O-glucoside
Relative Abundance
Time (min)
3.77 3.90 4.65 4.88 5.16 5.52 5.89

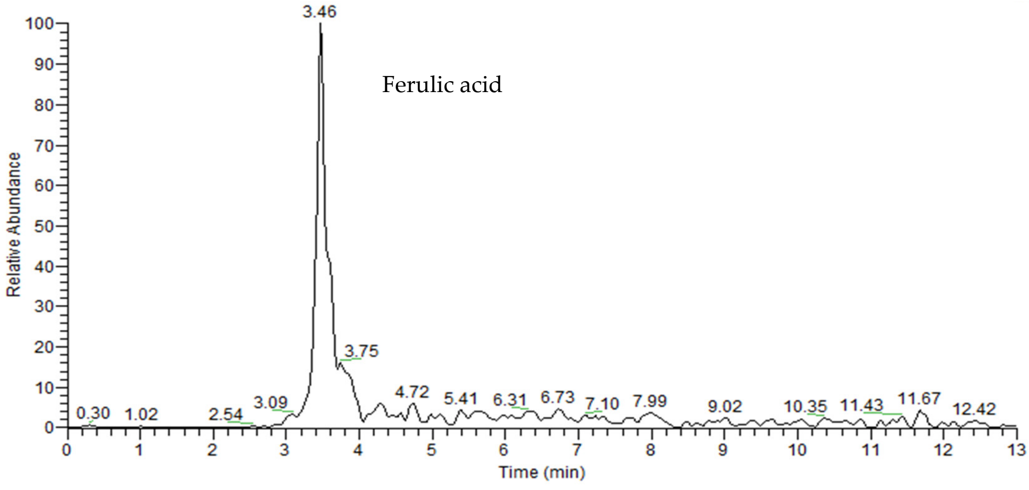
3.46
Ferulic acid
Relative Abundance
Time (min)
0.30 1.02 2.54 3.09 3.75 4.72 5.41 6.31 6.73 7.10 7.99 9.02 10.35 11.43 11.67 12.42

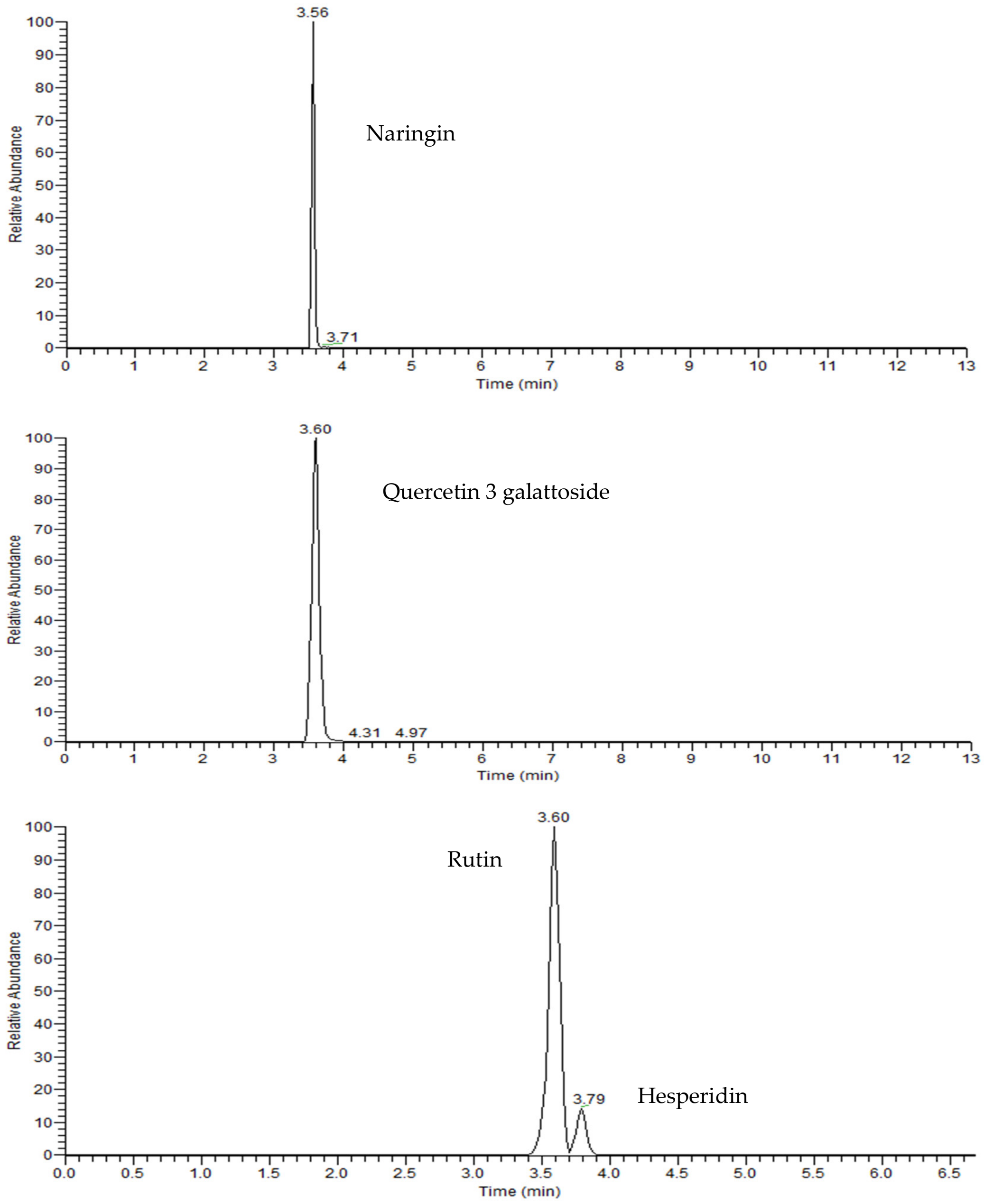

Naringin
3.56
3.71
Relative Abundance
Time (min)
Quercetin 3 galattoside
3.60
4.31
4.97
Relative Abundance
Time (min)
Rutin
3.60
Hesperidin
3.79
Relative Abundance
Time (min)

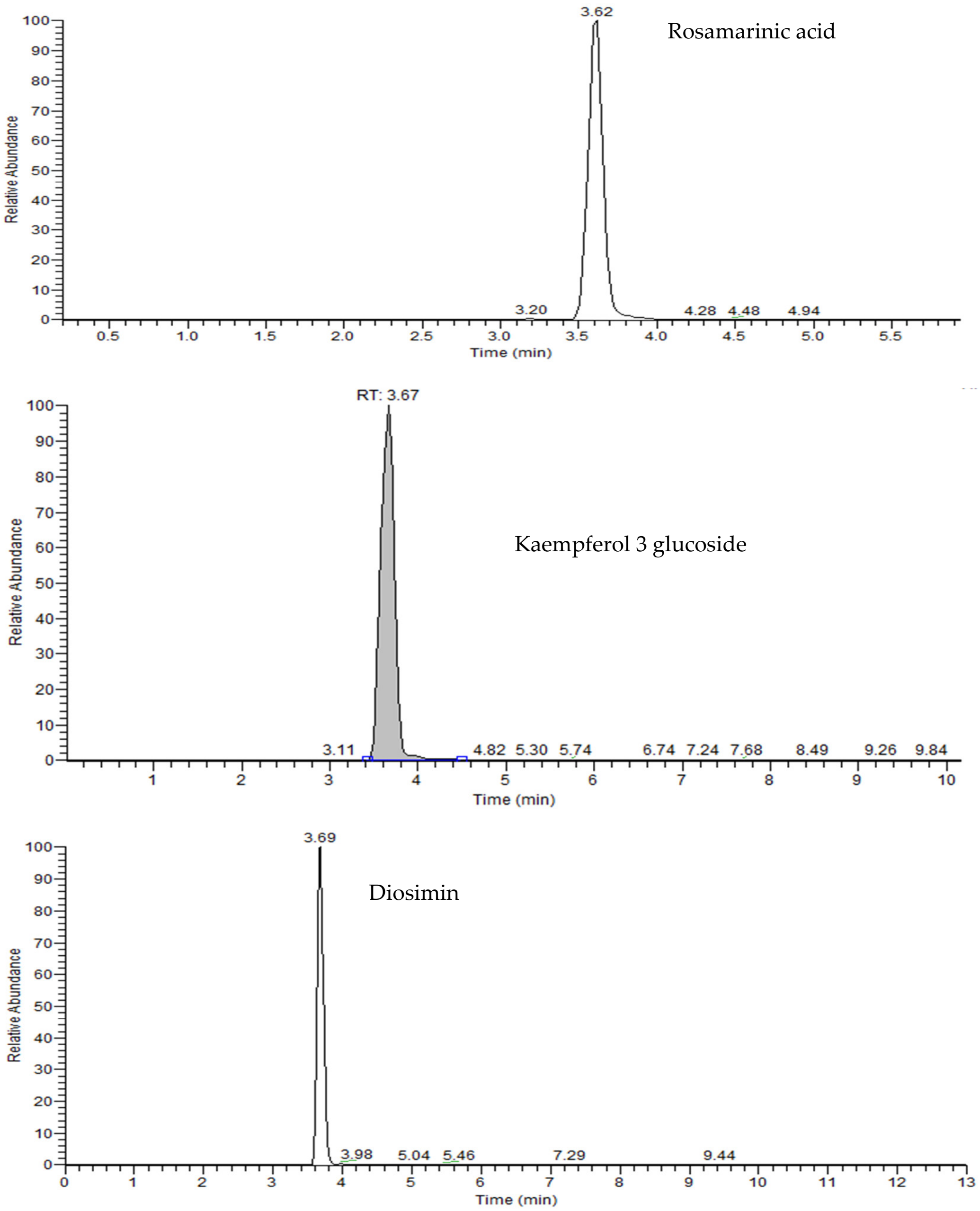
Rosamarinic acid
3.62
3.20
4.28
4.48
4.94
Relative Abundance
Time (min)
Kaempferol 3 glucoside
RT: 3.67
3.11
4.82
5.30
5.74
6.74
7.24
7.68
8.49
9.26
9.84
Relative Abundance
Time (min)
Diosimin
3.69
3.98
5.04
5.46
7.29
9.44
Relative Abundance
Time (min)

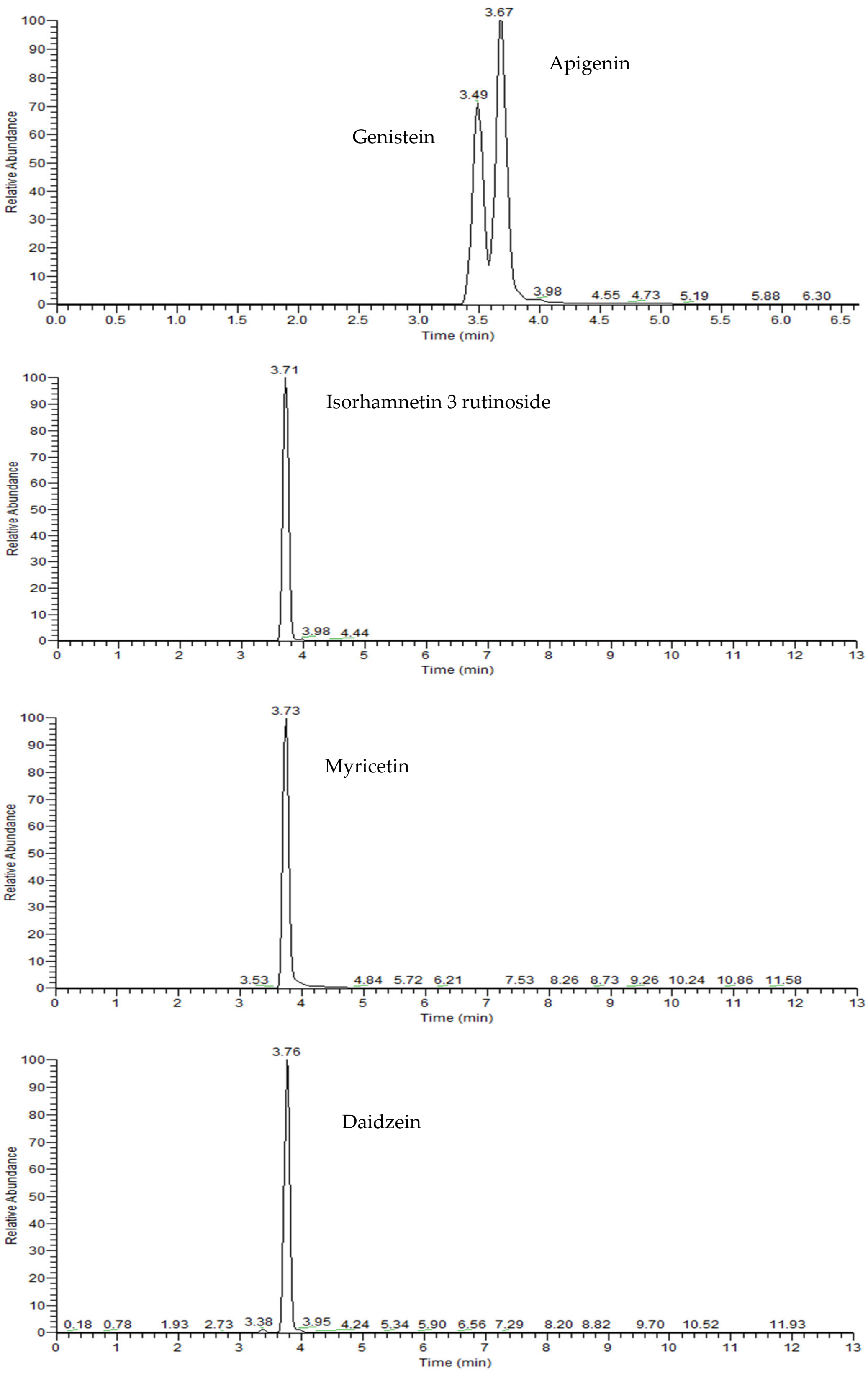
Genistein
Apigenin
3.49
3.67
Isorhamnetin 3 rutinoside
3.71
Myricetin
3.73
Daidzein
3.76
Relative Abundance
Time (min)

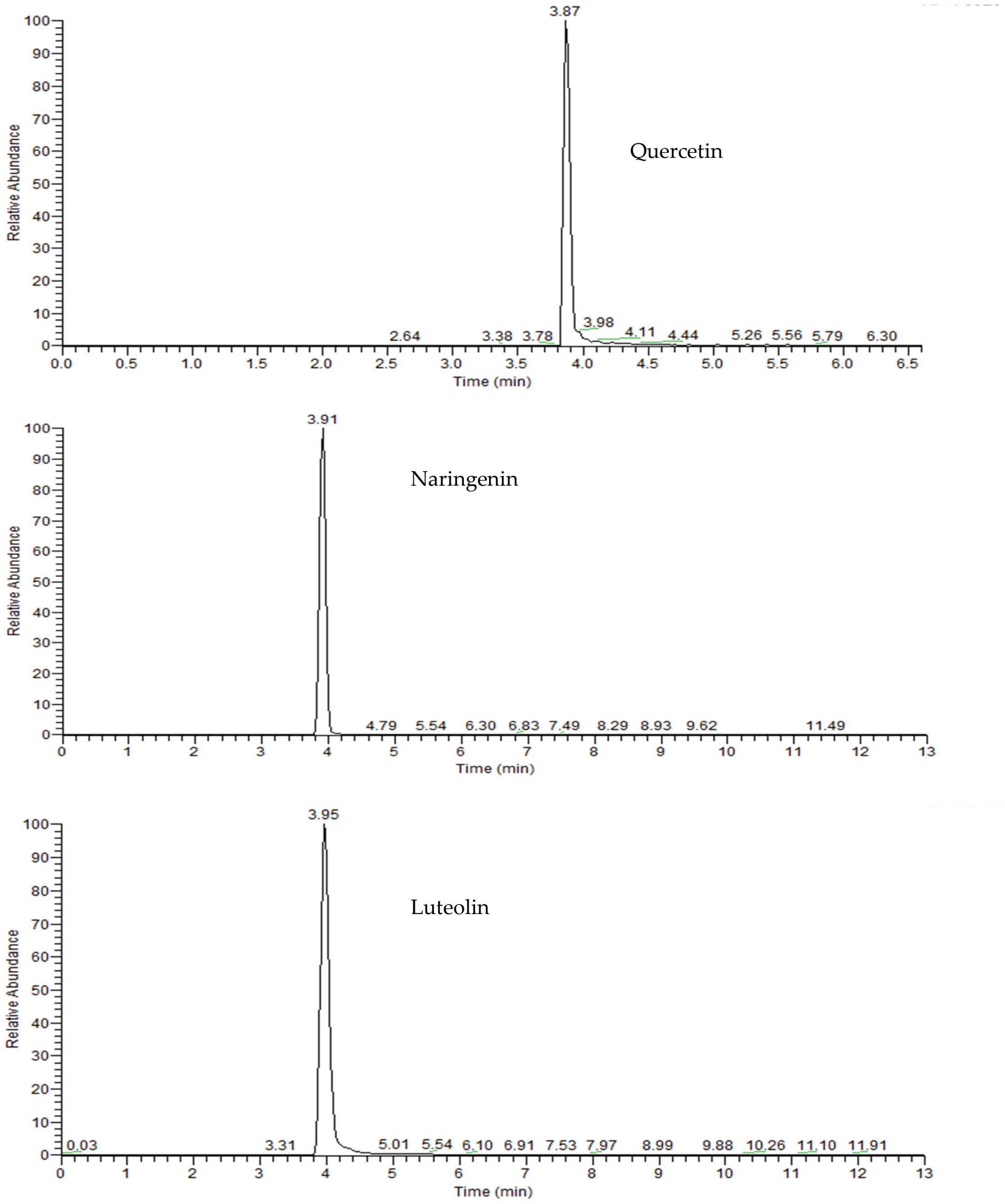

Figure S2. Plots of twenty-one representative extracted ion chromatograms.

Table S2. Total phenolic content in the CT-NARC and CT-ARC samples.

Samples	TPC mg GAE/g±SD	
Cellulose	0.2±0.00	
	**CT-NARC**	**CT- ARC**
Digestion Stage		
Oral stage	N.D.	N.D.
Gastric stage	N.D.	N.D.
Duodenal stage	N.D.	N.D.
Pronase E	N.D.	N.D.
Viscozyme L	N.D.	N.D.
Total colonic stage	N.D.	N.D.

Abbreviations: N.D.: not detected; CT-ARC: control acid-resistant capsules ; CT-NARC: control non-acid capsules.

Table S3. Bioaccessibility of polyphenolic compounds in not-encapsulated pea pods water-based extracts.

Sample	TPC mg GAE/g±SD
Not-encapsulated extracts	
Digestion Stage	
Oral stage	1.34±0.02
Gastric stage	1.07±0.04
Duodenal stage	1.54±0.05
Pronase E	1.66±0.04
Viscozyme L	1.48±0.06
Total colonic stage	3.14±0.05

Abbreviations: mg GAE/g: milligrams of gallic acid equivalent per gram of dry extract.

Table S4. Antioxidant capacity of not-encapsulated extracts evaluated by FRAP, DPPH, and ABTS assays during simulated GI digestion.

Sample	DPPH mmol/kg±SD	ABTS mmol/kg±SD	FRAP mmol/kg±SD
Not-encapsulated extract			
Digestion stage			
Oral stage	2.1 ± 0.1	1.8 ± 0.2	0.8 ± 0.1
Gastric stage	1.6 ± 0.1	1.6 ± 0.2	0.6 ± 0.1
Duodenal stage	1.7 ± 0.3	2..3 ± 0.2	1.1 ± 0.2
Pronase E stage	1.8 ± 0.2	3.0 ± 0.2	1.9 ± 0.4
Viscozyme L stage	1.5 ± 0.1	1.6 ± 0.3	1.2 ± 0.1
Total colonic stage	3.3 ± 0.2	4.6 ± 0.3	3.1 ± 0.3

Table S5. Correlation between TPC and data obtained by the FRAP, DPPH, and ABTS tests.

**Assay**	**Gastric Stage**	**Duodenal Stage**	**Pronase Stage**	**Viscozyme Stage**
	R^2	R^2	R^2	R^2
DPPH	0.98	0.98	0.98	0.97
ABTS	0.97	0.98	0.96	0.98
FRAP	0.97	0.97	0.97	0.97

The correlation coefficients were evaluated by using Pearson's method.

Table S6. Antioxidant activity evaluated by FRAP, DPPH, and ABTS assays in the CT-NARC and CT-ARC samples.

	**DPPH mmol/kg±SD**		**ABTS mmol/kg±SD**		**FRAP mmol/kg±SD**	
Cellulose not-digested	0.3 ± 0.0		0.2 ± 0.0		0.3 ± 0.0	
	**CT-NAR**	**CT- AR**	**CT-NAR**	**CT- AR**	**CT-NAR**	**CT- AR**
Digestion stage						
Oral stage	N.D.	N.D.	N.D.	N.D.	N.D.	N.D.
Gastric stage	N.D.	N.D.	N.D.	N.D.	N.D.	N.D.
Duodenal stage	0.1 ± 0.0	N.D.	0.1 ± 0.0	N.D.	N.D.	N.D.
Pronase E stage	N.D.	0.1 ± 0.0	N.D.	N.D.	0.1 ± 0.0	0.1 ± 0.0
Viscozyme L stage	N.D.	n.d.	N.D.	N.D.	N.D.	N.D.
Total colonic stage	N.D.	0.1 ± 0.0	N.D.	N.D.	0.1 ± 0.0	0.1 ± 0.0

Abbreviations: N.D.: not detected; CT-ARC: control acid-resistant capsules ; CT-NARC: control non-acid capsules.
